# Construction of a pathway to C_50_-ε-carotene

**DOI:** 10.1371/journal.pone.0216729

**Published:** 2019-05-14

**Authors:** Yusuke Otani, Takashi Maoka, Shigeko Kawai-Noma, Kyoichi Saito, Daisuke Umeno

**Affiliations:** 1 Department of Applied Chemistry and Biotechnology, Chiba University, Chiba, Japan; 2 Research Institute for Production Development, Kyoto, Japan; Huazhong University of Science and Technology, CHINA

## Abstract

Substrate tolerance of bacterial cyclases has been demonstrated in various contexts, but little is known about that of plant cyclases. Here, we tested two plant ε-cyclases to convert C_50_-lycopene, which we previously established by rounds of directed evolution. Unlike bacterial β-cyclases, two-end cyclase from lettuce exhibited complete specificity against this molecule, indicating that this enzyme has some mechanism that exerts size-specificity. *Arabidopsis* one-end cyclase At-y2 showed detectable activity to C_50_-lycopene. Interestingly, we found that it functions as a two-end cyclase in a C_50_ context. Based on this observation, a possible model for substrate discrimination of this enzyme is proposed.

## Introduction

**Fig 1:**
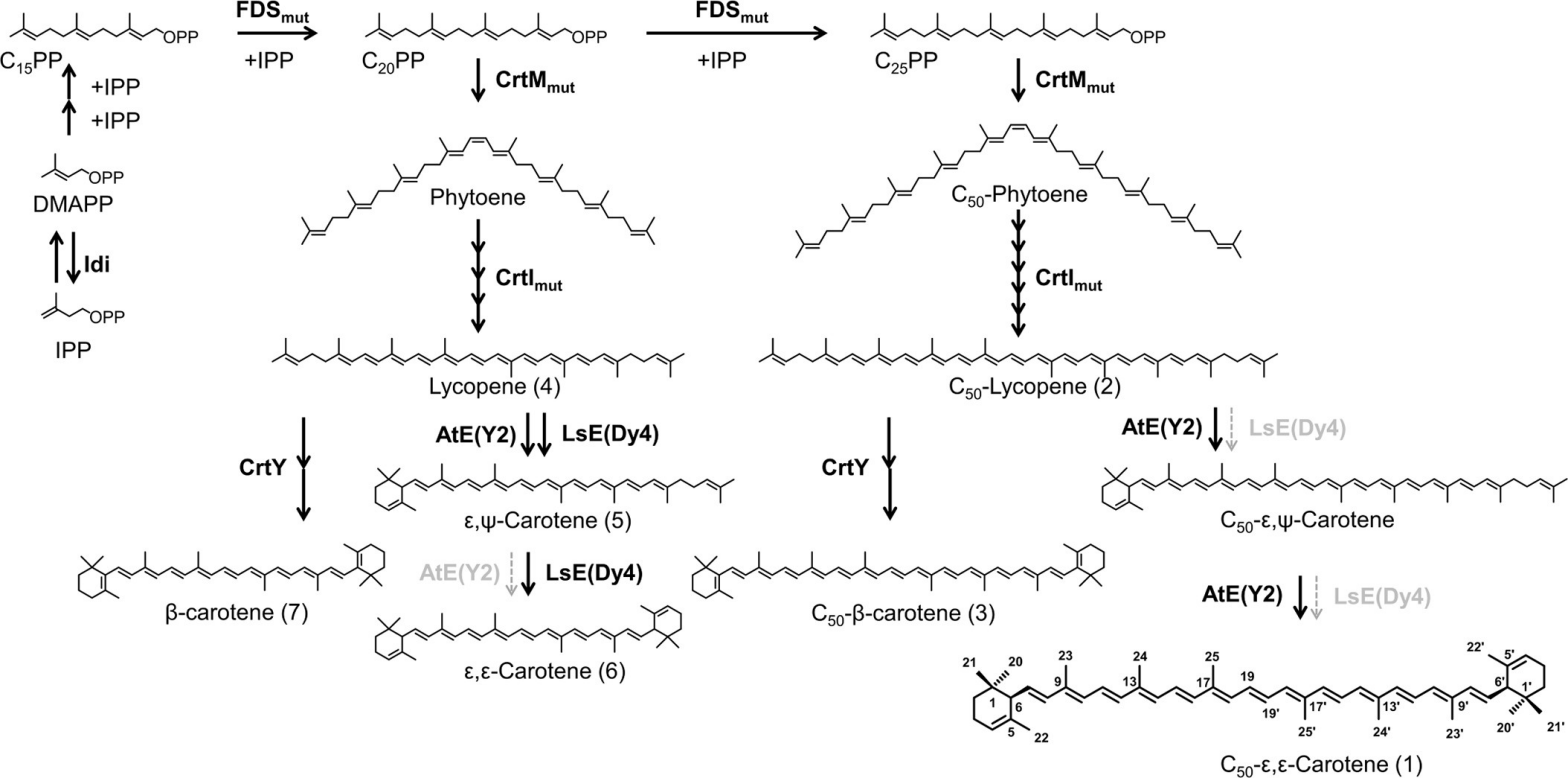
Pathways for C_50_-ε-cyclic–carotenoids.

Most of the natural cyclic carotenoids have β-cyclic end groups, but plants and algae biosynthesize carotenoids with ε-cyclic ends [1,2] (Fig 1). One of the two major ε-cyclic carotenoids seen in nature, α-carotene (β, ε-carotene), is superior to β-carotene (β, β-carotene) both as a singlet quencher [3] and as an antioxidant [4]. In addition, α-carotene has been shown to possess proactive skin-care functions [5,6]. Lutein, the hydroxylated form of α-carotene, is known to be a major macular pigment of humans [7], and its oral supplementation has been shown to have preventive and alleviative effects on cataracts [8–11] and age-related macular degeneration [12]. An LDL-lowering effect was also reported for lutein [13]. Given the unique biological functions and industrial value of ε-cyclic carotenoids, together with their expected pharmacokinetics distinct from those with provitamin-A activities [14], novel carotenoids with ε-end structures would be attractive targets for pathway engineers.

Interestingly, however, there are relatively few carotenoids with ε-cyclic ends in nature. Lettuce accumulates ε-carotene (ε, ε-carotene), and two-step lycopene ε-cyclase (named LsE) has been identified from this organism [15].Tuna and yellowtail accumulate tunaxanthin (2,2′-dihydroxylated ε-carotenoid) as one of the major carotenoids in their fins. Interestingly, however, it has been shown to be a zeaxanthin metabolite, not a product of the hydroxylation of ε-carotene [16]. It is not known how zeaxanthin is converted to tunaxanthin, and to the best of our knowledge, only one report has been published on the microbial production of rare/novel ε-cyclic carotenoids [17]. Here, promiscuous carotenoid synthase CrtM from *Staphylococcus aureus* was co-expressed with CrtE (geranylgeranyl diphosphate synthase) in *Escherichia coli*, resulting in the production of a phytoene-like compound with a C_35_ backbone. This asymmetrical carotenoid-like compound was successfully desaturated by *Pantoea ananatis* CrtI for three or four steps C_35_ lycopene, and then ε-cyclized by lettuce LsE and *Arabidopsis thaliana* AtE.

We recently engineered non-natural biosynthetic pathway toward C_50_-β-carotene by mixing, matching, and mutating the carotenogenic enzymes from the C_30_ and C_40_ carotenoid pathways. This pathway was demonstrated to be further extended by additionally expressing various decoration enzymes such as ketorase and hydroxylase, resulting in the efficient and selective production of C_50_-astaxanthin, C_50_-canthaxanthin, and C_50_-zeaxanthin. To further expand the carotenoid structures thus obtained, we decided to establish the pathway to C_50_-ε-carotenoids, which should provide a new starting point toward a variety of novel carotenoids. To this end, we additionally expressed ε-cyclases from two different sources, to the *E*. *coli* strain engineered to specifically produce C_50_-lycopene. Despite the end structures (ψ-ends) of C_50_ lycopene being identical to those of lycopene, lettuce two-step ε-cyclase (LsE) could not produce any cyclized compound. In contrast, monocyclic ε-cyclase from *Arabidopsis* (AtE) accepted C_50_-lycopene as a substrate. Interestingly, the resultant novel carotenoid had two ε-ends. Expression tuning, N-terminal truncation, and modification of cell growth medium resulted in the collection of >100 μg/g per dry cell weight (DCW) of C_50_-ε-carotene.

## Methods and methods

### Strains and reagents

*E*. *coli* XL10-Gold (Stratagene) was used for cloning, while XL1-Blue (Stratagene) was used for carotenoid production experiments. Cells were grown in Luria–Bertani (LB) Lennox medium for cloning, preculture, or plate cultivation and in TB medium for carotenoid production. Reagents used were 50 mg mL^−1^ carbenicillin (carb), 30 mg mL^−1^ chloramphenicol (cm), and 20% (w/v) L-arabinose, and were purchased from Nacalai Tesque (Kyoto, Japan).

### Plasmid construction

The plasmids and genes used in this study are listed in S1 Table and S2 Table, respectively. The plasmids pAC-*fds*_*Y81A*,*V157A-crtMF26A*,*W38A*,*F233S*_ and pAC-*fds*_*Y81M*_*-crtM*_*F26A*,*W38A*_ were derived from a previous study [18]. The plasmids for the downstream enzymes (CrtI, CrtY, AtE, and LsE) and their derivatives are based on the pUCara vector [18] with arabinose promoter.

pUCara-*crtI*_*N304P*_-*AtE* and pUCara-*crtI*_*N304P*_-*LsE* were created by inserting AtE or LsE into the ApaI-SpeI site of pUCara-crtI_N304P_-crtY in place of CrtY. The RBS score of AtE was calculated using RBS calculator[19,20] and modified using the following primer: 5′-TTTGGGCCCGGAAAGGAGGAACAAAATGGAGTGTGTTGGGGCTAG-3′.

### Culture conditions for carotenoids

Plasmids were transformed into XL1-Blue cells, which were then plated on LB-agar plates and incubated at 37°C for 24 h. For liquid culture experiments, colonies were inoculated into 2 ml of LB medium in culture tubes, which were shaken at 37°C for 16 h. The cultures were then diluted 100-fold into 40 ml of fresh TB medium in 200 ml flasks, and shaken for 24 h, followed by the addition of L-arabinose to a final concentration of 0.2% (w/v) and an additional 24 h of shaking. For plate culture experiments, fresh colonies were inoculated with 2 mL of LB medium in culture tubes, which were shaken at 37°C for 10 h. The cultures were then plated on LB agar plates covered with a nitrocellulose membrane (Pall, Port Washington, NY) and incubated at 37°C for 24 h. For colony color development, colonies on the nitrocellulose membrane were transferred to LB agar plates containing 0.2% (w/v) L-arabinose and incubated for an additional 12, 24, 48, or 72 h at room temperature. After cultivation, colonies were scraped off and collected and used for analysis.

### Carotenoid extraction and HPLC analysis

Each cell culture was centrifuged at 3,270 ×g and 4°C for 15 min. The cell pellets were washed with 10 ml of 0.9% (w/v) NaClaq and repelleted by centrifugation. Carotenoids were extracted by adding 10 ml of acetone followed by vigorous shaking. One milliliter of hexane and 35 ml of 1% (w/v) NaClaq were added, samples were centrifuged at 3,270 ×g for 15 min, and the carotenoid-containing hexane phase was collected. The hexane was then evaporated by a vacuum concentrator. The extracts were then dissolved in 100 μl of THF:methanol = 6:4 for HPLC analysis. An aliquot of 1–5 μl of the final extract was analyzed using a Shimadzu Prominence HPLC system (Shimadzu, Kyoto, Japan) equipped with a photodiode array detector and mass spectrometer. A Spherisorb ODS 2 column (150 × 2.1 mm I.D., 5 mm particles; Waters, Milford, MA) was used. The mobile phase was acetonitrile/tetrahydrofuran/methanol (38:4:58 v/v, 0.3 ml min^−1^). The detector used was a photodiode array (200–700 nm) and APCI-MS. The MS was operated in selected ion monitoring mode scanning for the molecular ion at 669.5 *m/z*, at an interface temperature of 300°C, DL temperature of 300°C, interface voltage of ±4,500 V, and neutral DL/Qarray, using N_2_ as nebulizing gas.

Individual carotenoids were quantified by their peak areas using a calibration curve generated with known amounts of β-carotene, then multiplying by the molar extinction coefficient (ε) of β-carotene (138,900 M^−1^ cm^−1^ at 450 nm) and dividing by the e value for the carotenoid in question. Production weights of carotenoids were then normalized to the DCW of each culture. The DCW was calculated using an OD_600_-DCW calibration curve.

### Apparatus

UV-visible (UV-VIS) absorption spectra were recorded with a Hitachi U-2001 spectrophotometer (Hitachi Field Navigator, Tokyo, Japan) in diethyl ether (Et_2_O). MS analysis of carotenoids was performed using a Waters Xevo G2S Q-TOF mass spectrometer (Waters Corporation, Milford, CT, USA). ESI-TOF-MS spectra were acquired by scanning from *m/z* 100 to 1,500 with a capillary voltage of 3.2 kV, cone voltage of 40 eV, and source temperature of 120°C. The ^1^H-NMR (500 MHz) spectrum was measured with a Varian Unity Inova 500 spectrometer (Varian Corporation, Palo Alto, CA, USA) in CDCl_3_ with TMS as an internal standard. Because of the small amount of carotenoid sample (about 30 μg), ^1^H-NMR was measured using a SHIGEMI microtube (sample solution volume 200 μl) (Shigemi Co., Ltd., Tokyo, Japan). The CD spectrum was recorded in Et_2_O at room temperature with a Jasco J-500C spectropolarimeter (JASCO Corporation, Tokyo, Japan). Preparative HPLC was performed with a Hitachi L-6000 HPLC intelligent pump and Hitachi L-4250 UV-VIS detector (Hitachi Field Navigator, Tokyo, Japan) set at 450 nm. The column used was a 250 × 4.6 mm i.d. Cosmosil 5C18-II (Nacalai Tesque, Kyoto, Japan).

## Results

### C_50_ functions of lycopene ε-cyclases

In an attempt to introduce an ε-ring in C_50_-carotenoids in *E*. *coli*, we tested two different ε-cyclases: one-ring cyclase from *Arabidopsis thaliana* [21] (AtE) and two-ring cyclase from *Lactuca sativa* (Roman lettuce) (LsE), using an expression construct previously established for C_50_-β-carotene [18]. The genes were inserted downstream of the desaturase mutant gene (*crtI*_*N304P*_) of pUC-araP-*crtI*_*N304P*_ (pMB1-based vector for expressing CrtI_N304P_ under an arabinose promoter [18]), yielding pUCara-*crtI*_*N304P*_-AtE and pUCara-*crtI*_*N304P*_-LsE, respectively. Cells harboring the plasmid for C_50_-phytoene biosynthesis (pAC-pacP-*fds*_*Y81A*,*V157A*_-*crtM*_*F26A*,*W38A*,*F233S*_ [18]) were additionally transformed with these plasmids, and the transformant cells were grown in liquid Terrific Broth (TB) medium. Owing to the toxicity and instability of C_50_-lycopene, *crtI*_*N304P*_ has to be induced in the later stage (at 24 h after seeding) of growth to achieve efficient production of C_50_-carotenoids [18]. After an additional 24 h of shaking, the carotenoid fraction was acetone-extracted from the cell for HPLC analysis.

**Fig 2:**
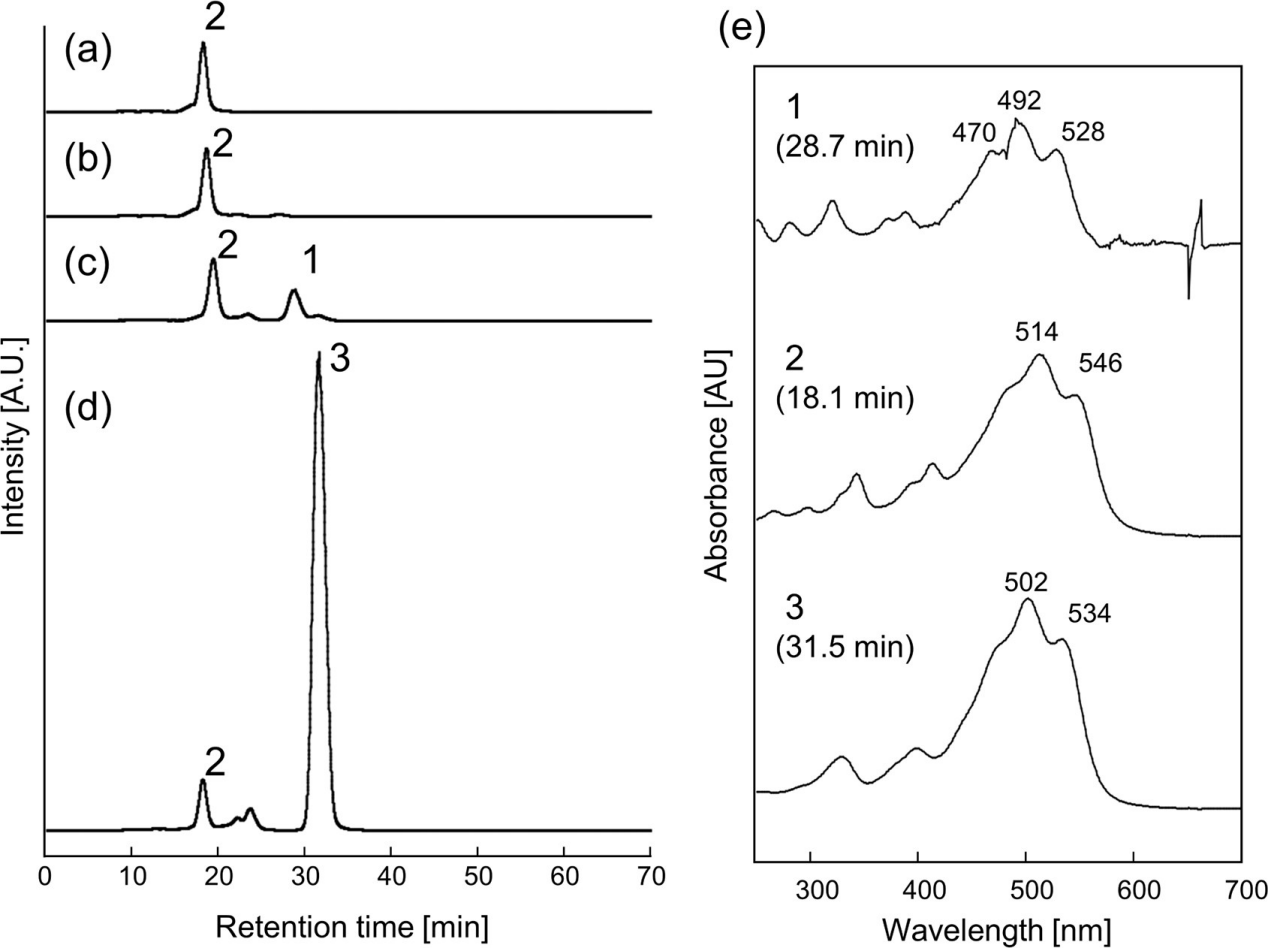
Activities of *Arabidopsis* (AtE) and lettuce (LsE) cyclases on C_50_-lycopene. *E*. *coli* harboring pAC-pacP-*fds*_*Y81A*,*V157A*_-*crtM*_*F26A*,*W38A*,*F233S*_ and (a) pUCara-*crtI*, (b) pUCara-*crtI*_*N304P*_-*LsE*, (c) pUCara-*crtI*_*N304P*_-*AtE*, (d) pUCara-*crtI*-*crtY*. The absorption spectrum of each peak is shown in (e).

**Fig 3:**
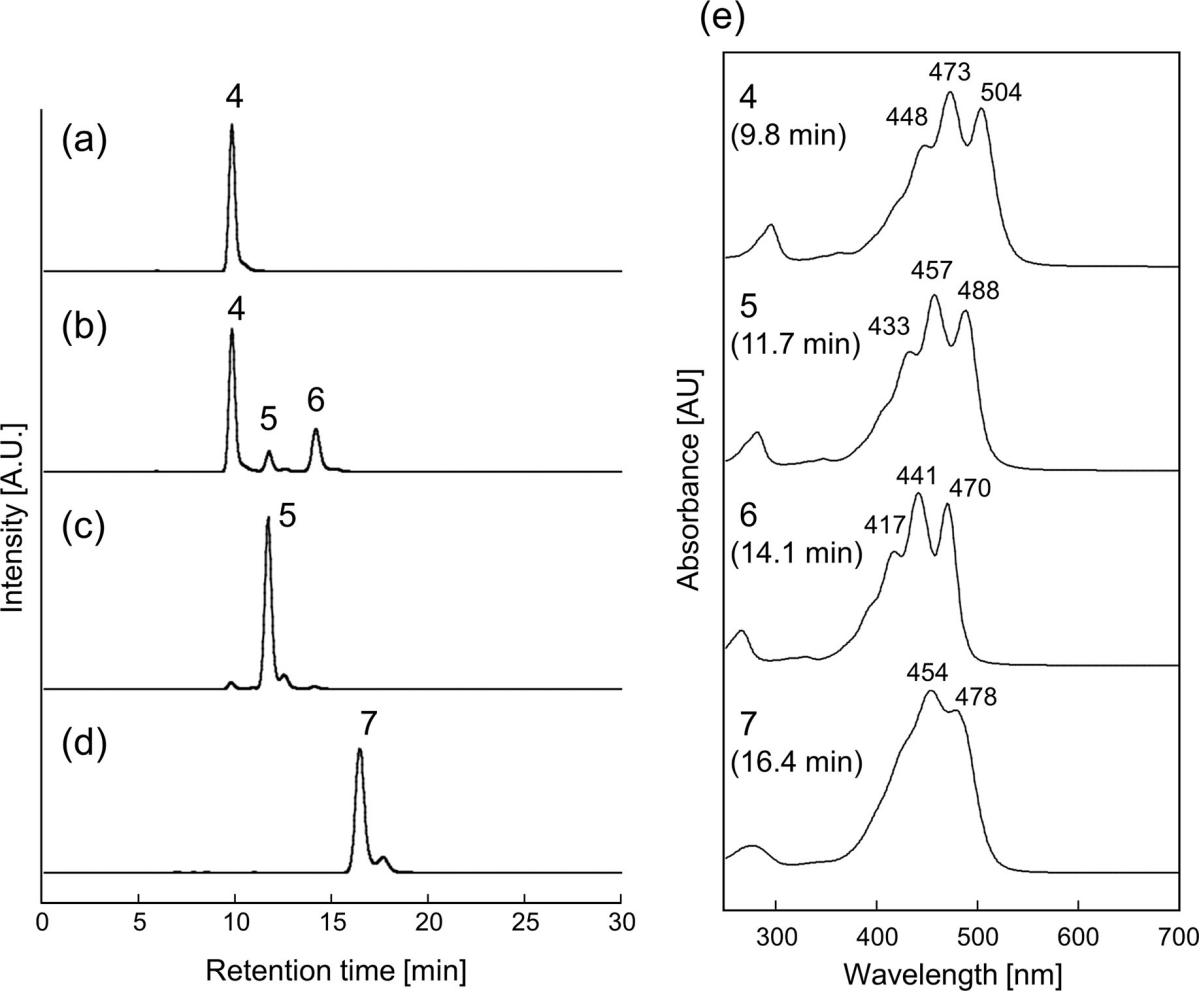
Activities of e-cyclase from *Arabidopsis* (AtE) and lettuce (LsE) on C_40_-(natural) lycopene. *E*. *coli* harboring pAC-pacP-*fds*_*Y81A*_-*crtM*_*F26A*,*W38A*_ and (a) pUCara-*crtI*, (b) pUCara-*crtI*-*LsE*, (c) pUCara-*crtI*-*AtE*, (d) pUCara-*crtI*-*crtY*. The absorption spectrum of each peak is shown in (e).

With this construct and protocol, bacterial β-cyclase CrtY efficiently converted C_50_-lycopene, resulting in the accumulation of 900 μg of β-cyclic C_50_ carotenoids per DCW (Fig 2D). In contrast, we did not observe any new peak by expressing LsE (Fig 2B). This result could have been partly due to the insufficient heterologous *E*. *coli* activity of LsE. Indeed, even its cognate substrate C_40_-lycopene was not fully converted to ε, ε-carotene (Fig 3B) with our expression system. Note that this result does not deny the possibility of cyclization of C_50_-lycopene by LsE in any conditions. By careful search for right working environment, by elevating expression level, or by adding appropriate mutations, LsE could exhibit some detectable cyclase activity on C_50_ substrate. When AtE was co-expressed with genes for C_50_-lycopene synthesis, we observed one new peak (Peak 1 in Fig 2C) with a molecular mass of 668.5, identical to that of C_50_-lycopene. Note that this enzyme fully consumed its natural substrate C_40_-lycopene and yielded ε, ψ-carotene as the sole product (Fig 3C).

The absorption spectrum of **compound 1** had three peaks (470, 492, and 528 nm), which were significantly blue-shifted from those of C_50_-lycopene (514 and 546 nm, polyene with 15 conjugated double bonds) or β-C_50_ carotene (502 and 534 nm, 13 conjugated double bonds with two ring-derived ones: 5–6 and 5′-6′). From the preservation of the fine structure in the absorption spectrum and the extent of the blue shift (492 nm from 514 nm), together with the retention time in reverse-phase HPLC (i.e., higher hydrophobicity), we assumed the **1** should be either C_50_-delta-(ψ,ε)-carotene (with 14 conjugated double bonds) or C_50_-ε-carotene (with 13 conjugated double bonds). Note that the absorption spectrum of **1** was nearly identical to that of the natural carotenoid 3,4-didehydrolycopene [22], which also possesses a linear polyene chromophore with 13 conjugated double bonds.

### Metabolic engineering of ε-cyclic C_50_ carotenoid pathway

**Fig 4:**
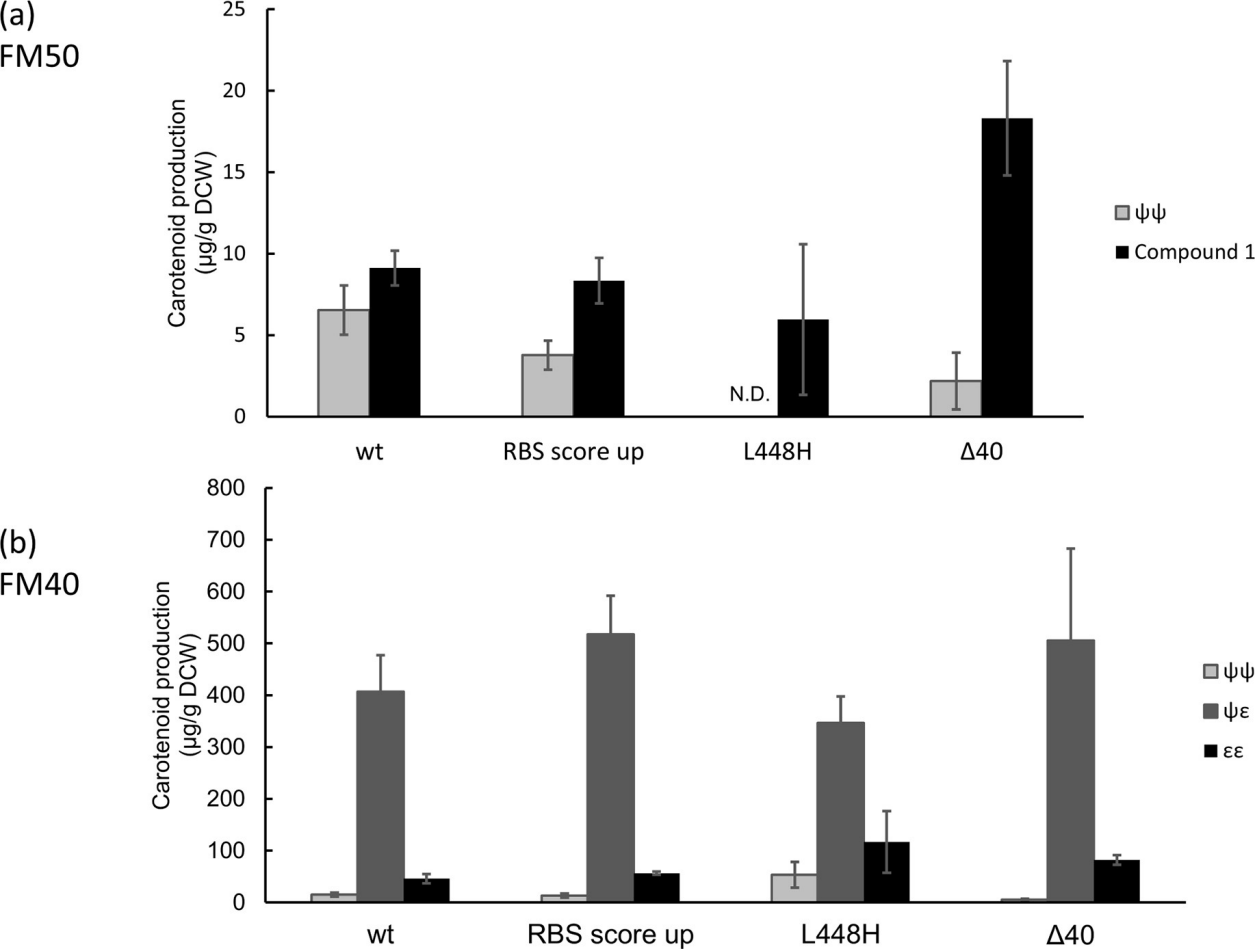
Metabolic engineering of the pathway to ε-cyclic C_50_-carotenoid. Indicated plasmids were introduced into *E*. *coli* harboring either (a) pAC- pacP-*fds*_*Y81A*,*V157A*_-*crtM*_*F26A*,*W38A*,*F233S*_ or (b) pAC-pacP-*fds*_*Y81A*_-*crtM*_*F26A*,*W38A*_ and the accumulated carotenoid was extracted from the resultant transformants. The data are presented as the average of three independent experiments, with error bars representing the standard deviation from the mean.

The low yield of this compound with initial constract discouraged us from determining the structure of this ε-cyclic C_50_ carotenoid. Instead, we decided to improve its production level by metabolic engineering. We initially hypothesized that the low yield of **1** simply arises from the poor expression/activity of AtE. This is because *P*. *ananatis* CrtY can produce far more C_50_-β-carotene (~800 μg/g-DCW) with the same expression conditions, and the supply of substrate (C_50_-lycoepene) cannot be the limiting factor. Therefore, we attempted to elevate the transcription initiation efficiency, thereby increasing the expression level of this cyclase (RBS score was elevated from 1274.52 to 18,732.17) (AtE RBS score up in Fig 4A). However, we did not observe any detectable increase in the level of 1. This change also did not alter the product distribution in the C_40_ pathway (Fig 4B).

Previously, Cunningham *et al*. reported that AtE could be converted into two-step ε-cyclase only by one amino acid substitution (L448H) [15]. We thought that this specificity-relaxing mutation could also increase the promiscuity toward a large substrate (C_50_-lycopene), thereby increasing the production of **1**. However, we observed no increase in the level of **1**. Interestingly, with our construct, we observed a very subtle effect (if not none) of L448H also in the natural C_40_ pathway (Fig 4B).

Next, we created several N-truncated mutants of AtE (those with the first 27, 40, and 70 residues removed) and analyzed them with the aim of improving the production of **1**. Removal of N-terminal domains is a popular and proven strategy to improve the performance/activity of isoprenoid enzymes (as has been shown for squalene synthase [23,24] and monoterpene synthase [25], for example) in heterologous hosts. Indeed, the expression of AtE_Δ40_ led to the accumulation of twice as much of **1** as in the cells expressing full-length AtE (Fig 4A). Still, the level of 1 remained 15 μg/g-DCW. This is about 3% of that for δ-carotene (Fig 4B).

**Fig 5:**
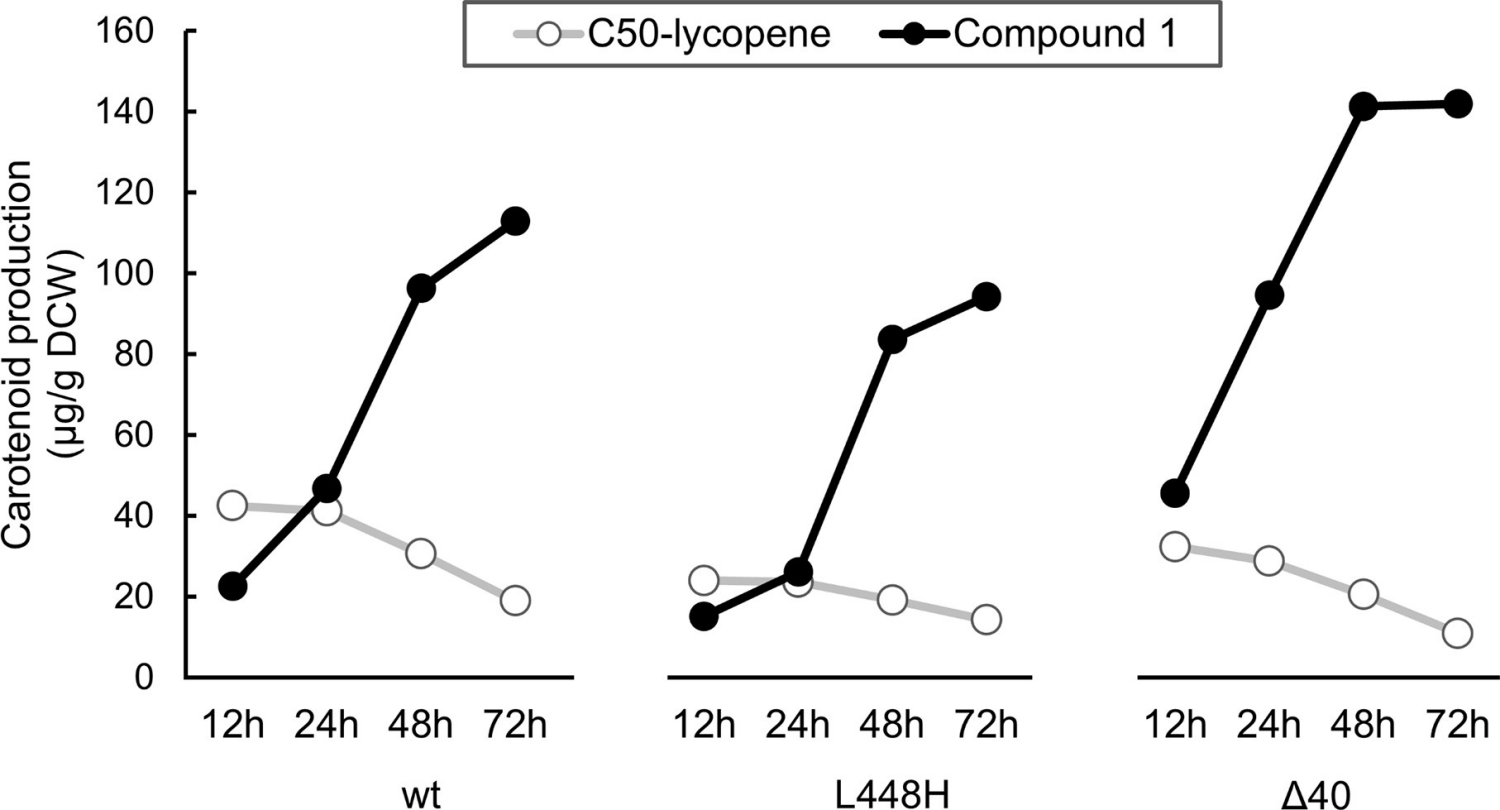
Production of ε-cyclic C_50_ carotenoid (1). *E*. *coli* expressing WT-AtE, AtE(L440H), or AtE(Δ40) was transformed with pAC-pacP-*fds*_*Y81A*,*V157A*_-*crtM*_*F26A*,*W38A*,*F233S*_ and the resultant transformant cells were grown on an LB-agar plate. The cells were suspended in medium, centrifuged, and acetone-extracted at different time points to measure the pigmentation level.

Among the factors that we tested, the most effective strategy to increase the production level of compound **1** was adjustment of the growth conditions. In liquid medium, transformants accumulated only a trace level of **1** in all tested conditions (see above), while the cells on the agar plate accumulated a much higher level of **1**. Forty-eight hours after induction on a TB-agar plate, the cells harboring AtE_Δ40_ accumulated 120 μg/g-DCW of **1** (Fig 5).

### Structural determination of ε-cyclic C_50_-carotenoid

Given the decent production level achieved by the aforementioned process, a reasonable amount (>50 μg) of compound **1** was isolated. **1** was purified by HPLC with an ODS column as stationary phase and chloroform:methanol (1:3) as mobile phase (S1 Fig). The molecular formula was determined to be C_50_H_68_ (*m/z* 668.5329 M^+^, calcd for C_50_H_68_, 668.5321) using positive ion Electro-spray ionization (ESI) time-of-flight (TOF) MS (ESI-TOF-MS) spectral data (S2 Fig). 1 showed absorption maxima at 490, and 519 nm in ether (S2 Fig). Characteristic ^1^H-NMR (S3 Fig) of H_3_-20, 20’ (δ 0.91), H_3_-21, 21’ (δ 0.83), H_3_-22, 21’ (δ 1.59), H-2,2’ (δ 1.46), H-2,2’ (δ 1.18), H-3, 3’ (δ 2.00), H-4, 4’ (δ 5.41), and H-6, 6’ (δ 2.19) indicated the presence of an ε-end group on both sides of the polyene chain [26]. The ^1^H-NMR signals of polyene parts (H-7, 7’ to H-19, 19’, H-23, 23’ to H-25, 25’) were assigned from COSY (S4 Fig), NOESY (S5 Fig), and ROESY (S6 Fig) spectral data, as shown in Table 1. From these spectral data, this compound was determined to be C_50_-ε, ε-carotene. The chirality at C-6, 6’ was determined to be an *R* configuration by CD spectral data (S7 Fig). As well as C40-(6*R*,6’*R*)-ε, ε-carotene, the CD spectrum of the compound showed a positive Cotton effect around 270 nm (Δε + 7.0 at 270 nm and Δε + 16.0 at 245 nm) [27].

**Table 1:** ^1^H NMR of ε, ε-carotene (1) in CDCl_3_.

	C_50_-ε, ε-carotene		
Position	δ	mult.	J (Hz)
H-2 (2') α	1.46	m	
H-2 (2') β	1.18	m	
H-3(3')	2.00	m	
H-4 (4')	5.41	br.s	
H-6 (6')	2.19	d	10
H-7 (7')	5.53	dd	15.5, 10
H-8 (8')	6.11	d	15.5
H-10 (10')	6.13	d	11
H-11 (11')	6.61	dd	15, 11
H-12 (12')	6.35	d	15
H-14 (14')	6.23	d	11
H-15 (15')	6.64	dd	15, 11
H-16 (16')	6.38	d	15
H-18 (18')	6.28	br. d	10
H-19 (19')	6.64	m	
H_3_-20 (20')	0.91	s	
H_3_-21 (21')	0.83	s	
H-22 (22')	1.59	d	1.5
H-22 (22')	1.92	s	
H_3_-23 (23')	1.97	s	
H_3_-24 (24')	1.99	s	
H_3_-25 (25')	1.99	s	

## Discussion

Substrate promiscuity of carotenoid-modifying enzymes is a key feature for quickly accessing novel carotenoid structures. Most of the carotenoid-modifying enzymes tested so far turned out to be remarkably tolerant to non-cognate substrates as long as they share the particular structural motif with the cognate ones [28]. For instance, bacterial lycopene β-cyclases (CrtY) were shown to be capable of cyclizing various acyclic carotenoids with so-called Ψ-ends without engineering. Included in these are neurosporene [21], ζ-carotene [29], C_30_-neurosporene [30], and C_35_- [17] and C_50_-lycopenes [18], yielding various novel carotenoids. Therefore, we initially expected that the two cyclases tested in this work (LsE and AtE) should cyclize C_50_-lycopene as well. Interestingly, however, LsE turned out to be completely inactive toward C_50_-lycoepne, and AtE exhibited detectable but very weak activity toward it. Although we eventually succeeded in biosynthesizing a reasonable amount of C_50_-ε-carotene (**1**), the maximum yield that we achieved was only *ca*. 20% of that for C_50_-β-carotene (**Fig 2**). To achieve a more efficient and selective production of this unique carotenoid, it should be necessary to test other classes of ε-cyclases or alter the product specificity of the β-cyclases exhibiting high activity toward C_50_-lycopene [18].

Most plants and algae accumulate β-carotene (β, β-carotene) and α-carotene (β, ε-carotene), but not ε-carotene (ε, ε-carotene), by sorting the pool of the common precursor lycopene in a biased manner. Most of the β-cyclases, including those involved in the biosynthesis of asymmetric α-carotene, are known to be two-step enzymes and can act on both ends of the lycopene, due to their “locally specific” nature: they accept substrates by precisely but solely recognizing the Ψ-end but not the entire structure. In contrast, most of the ε-cyclases are one-step enzymes: they act only on one side of lycopene, and once the ε-cyclic end is formed on one side, the other Ψ-end of the resultant δ-carotene (ε, Ψ-carotene) remains non-cyclized. In this way, plants ensure the production of β-carotene and α-carotene without accumulating the wasteful ε-carotene (ε, ε-carotene). However, it is not known exactly how ε-cyclases avoid acting on the Ψ-ends of the monocyclic carotenoids.

Lettuce has been shown to accumulate ε-carotene. Cunningham Jr. *et al*. cloned ε-cyclase from it and demonstrated that it is indeed a two-step enzyme [15]. By extensive chimeragenesis and mutagenesis, they identified the precise residue that determines the step number of ε-cyclases: mutation at 448 in AtE converted it into a two-step cyclase, and mutation at the corresponding residue (H457L) converted LsE into a one-step ε-cyclase. Similar was reported for β-carotene hydroxylase [31]. This enzyme acts on both sides of β-carotene to make zeaxanthin, but it becomes a one-step enzyme upon truncation of 129 amino acids at the N-terminus. It was claimed that this enzyme is homodimeric and elimination of its N-terminus abolished the dimer-forming capability, thereby converting it into a single-step enzyme. This dimer/monomer switching could be the mechanism for altering/tuning the apparent step number of ε-cyclases, as is claimed by the authors of these papers, but there is no further experimental support of this working hypothesis.

Another fruitful approach to elucidating the mechanism of step number control of carotene cyclases is to feed non-cognate substrates to the cyclase in question. Maresca *et al*. reported that β-cyclase CruA from green sulfur bacterium (*Chlorobium phaeobacteroides*) can act only on the one Ψ-end of lycopene, but not on that of γ-carotene (β, Ψ-carotene) [32]. In contrast, *C*. *phaeobacteroides* CruB acts on the Ψ-end of γ-carotene, but not on either side of lycopene. Interestingly, they discovered that CruB from *Chlorobaculum tepidum* acts as a two-step cyclase when neurosporene (7,8-unsaturated lycopene) was fed as the substrate. It was also shown that CruB from *C*. *phaeobacteroides* can act as a two-step cyclase on neurosporene. It was proposed that the 7,8-unsaturated Ψ-end cannot reach the active site of the cyclases due to its rigid and extended backbone, while the 7–8 saturated carotenoid can fold into the proper conformation that allows the other end to be placed into the active site.

**Fig 6:**
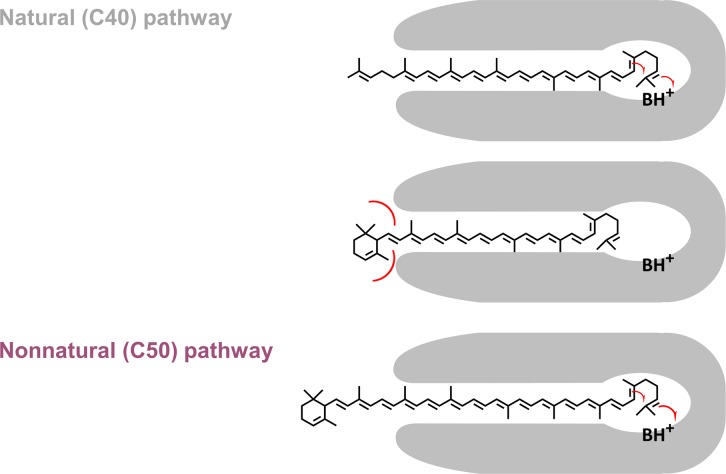
Possible mechanism for one-ring/two-ring determination by At-Y2.

In this paper, we present another example of context-dependent specificity switching well explained by this “ruler mechanism.” Two-step ε-cyclase LsE could equally and efficiently act on both sides of C_40_-lycopene (Fig 3B), while it failed to convert any side of C_50_-lycopene (Fig 2B). On the other hand, AtE cyclized both ends of the C_50_-lyopene (Fig 2C), while it clearly behaved as a one-end cyclase in the C_40_ pathway (Fig 3C). This is also despite their activity being markedly low. This behavior of AtE might be explained by the “ruler mechanism” (Fig 6). Here, the substrate (Ψ-end of carotenoids) must be properly inserted deeply into the reaction cavity to reach the catalytic center, as was recently shown for phytoene desaturase [33]. It is probable that the Ψ-end of monocyclic carotenoids (γ-carotene and δ-carotene) cannot reach the active site because the cyclized end of the C_40_-carotenoid crushes the residues forming the narrow substrate entrance. This negative design toward already-cyclized carotenoid does not apply to C_50_-carotenenoids: owing to the extended backbones, they can fully insert their Ψ-end without sterically crushing the other side with entrance. It is likely that the previously reported step number altering mutation (L448H) [15] might be located at the very entrance of the reaction cavity of this enzyme, thereby changing the extent of blocking for cyclic C_40_ carotenoids. It would be interesting to study whether CruAs and CruBs act as two-step cyclases to C_50_ lycopene and other non-cognate substrates might provide useful tools for testing the model of substrate discrimination of these enzymes as well as other carotenoid-modifying enzymes.

## Supporting information

S1 Table Plasmids used in this study. (DOCX)

S2 Table Genes used in this study. (DOCX)

S1 Fig HPLC diagram of C_50_-ε-carotene. (JPG)

S2 Fig TOF MASS and UV-VIS absorbance spectrum of C_50_-ε-carotene. (JPG)

S3 Fig ^1^H NMR spectrum of C50-ε-carotene. (JPG)

S4 Fig COSY spectrum of C50-ε-carotene. (JPG)

S5 Fig NOESY spectrum of C50-ε-carotene. (JPG)

S6 Fig ROESY spectrum of C50-ε-carotene. (JPG)

S7 Fig CD spectrum of C50-ε-carotene. (JPG)
