# Supplementary material for: Construction of a pathway to C50-ε-carotene
Source: PLoS One. 2019 May 14;14(5):e0216729. doi: 10.1371/journal.pone.0216729 (PMC6516660; doi:10.1371/journal.pone.0216729)
Supplement: S1 Table — (DOCX) [file pone.0216729.s001.docx]

| **Plasmid name** | **Vector** | **Reference** |
| --- | --- | --- |
| pAC-*fds_Y81A,V157A_*-*crtM_F26A,W38A,F233S_* | pACmod | [18] |
| pAC-*fds_Y81M_*-*crtM_F26A,W38A_* | pACmod | [18] |
| pUCara-*crtI_N304P_* | pUCara | [18] |
| pUCara-*crtI_N304P_-crtY* | pUCara | [18] |
| pUCara-*crtI_N304P_-AtE* | pUCara | This study |
| pUCara-*crtI_N304P_-LsE* | pUCara | This study |
