# Supplementary material for: Construction of a pathway to C50-ε-carotene
Source: PLoS One. 2019 May 14;14(5):e0216729. doi: 10.1371/journal.pone.0216729 (PMC6516660; doi:10.1371/journal.pone.0216729)
Supplement: S2 Table — (DOCX) [file pone.0216729.s002.docx]

| **Gene** | **encoding enzyme** | **Source** |
| --- | --- | --- |
| *fds_Y81A,V157A_* | C_25_PP synthase | *Geobacillus stearothermophillus* *fds* mutant |
| *fds_Y81M_* | C_20_PP synthase | *G. stearothermophillus* *fds* mutant |
| *crtM_F26A,W38A_* | C_35-50_ phytoene synthase  (C_40_ in this study) | *Staphylococcus aureus* *crtM* mutant |
| *crtM_F26A,W38A,F233S_* | C_35-55_ phytoene synthase  (C_50_ in this study) | *S. aureus* *crtM* mutant |
| *crtI_N304P_* | C_40-50_ phytoene desaturase | *Pantoea ananatis* *crtI* mutant |
| *crtY* | Lycopene β-cyclase | *P. ananatis* |
| *AtE* | Lycopene ε-cyclase | *Arabidopsis thaliana* |
| *LsE* | Lycopene ε-cyclase | *Lactuca sativa* |
