# Supplementary figures and images for: Construction of a pathway to C50-ε-carotene
Source: PLoS One. 2019 May 14;14(5):e0216729. doi: 10.1371/journal.pone.0216729 (PMC6516660; doi:10.1371/journal.pone.0216729)

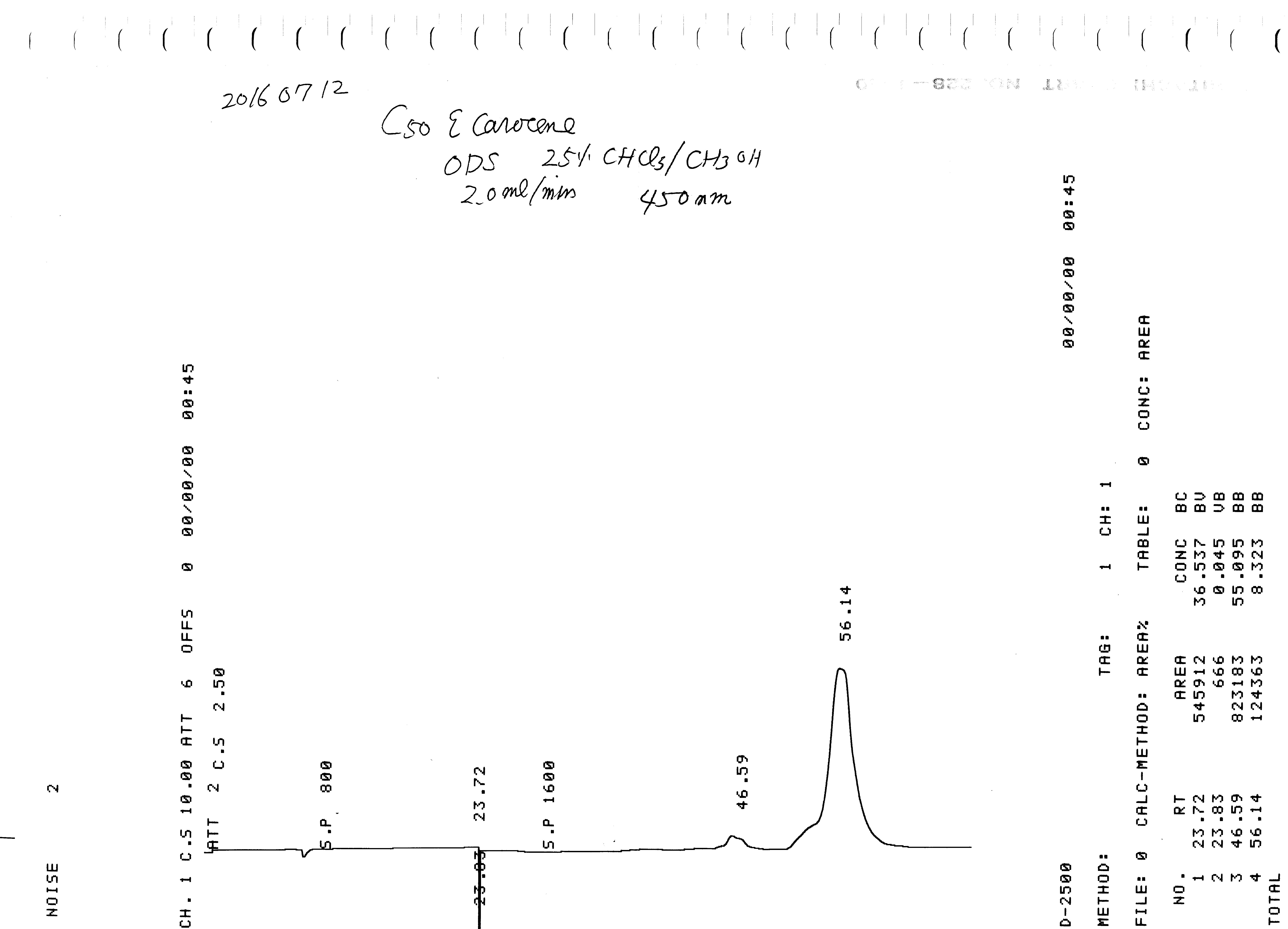

Supplement: S1 Fig — (JPG) [file pone.0216729.s003.jpg]

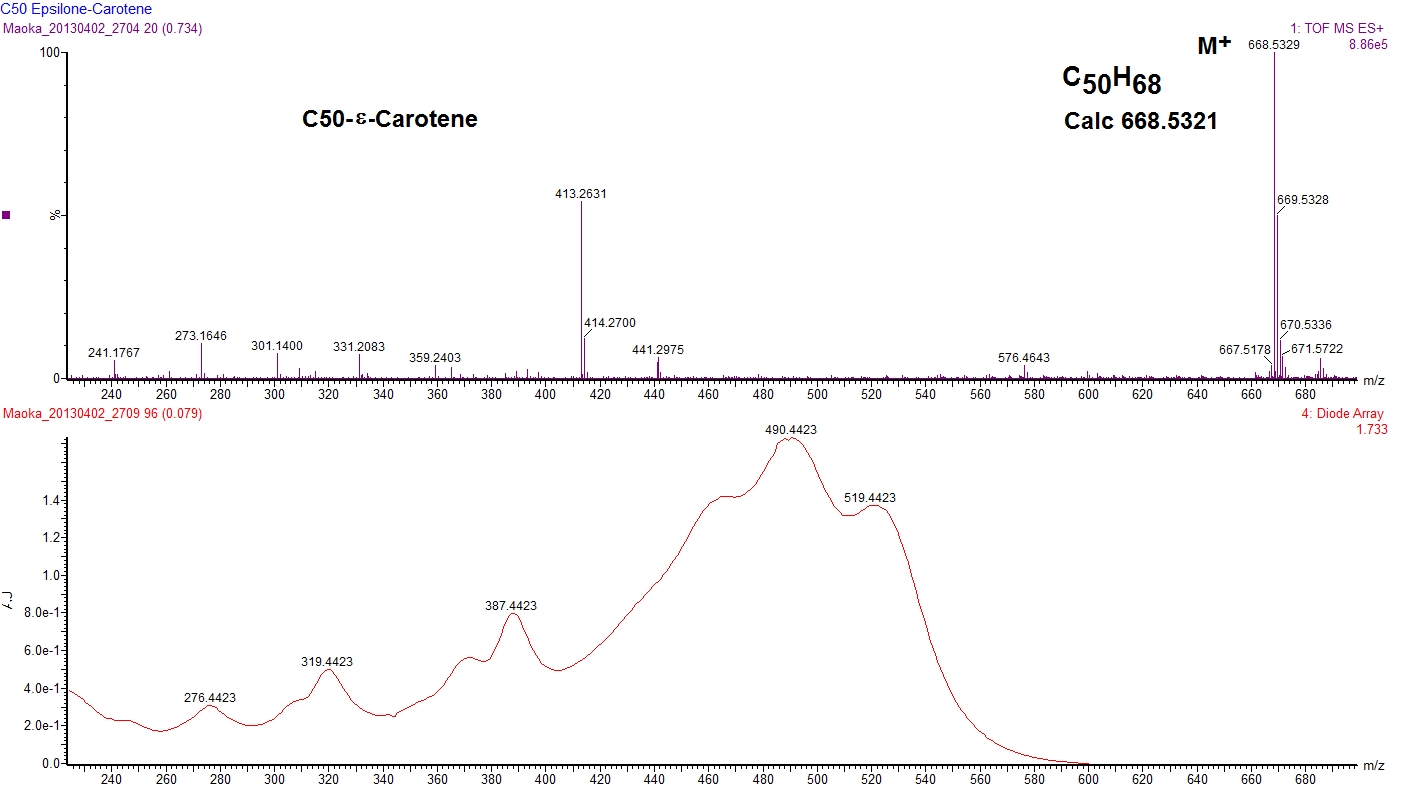

Supplement: S2 Fig — (JPG) [file pone.0216729.s004.jpg]

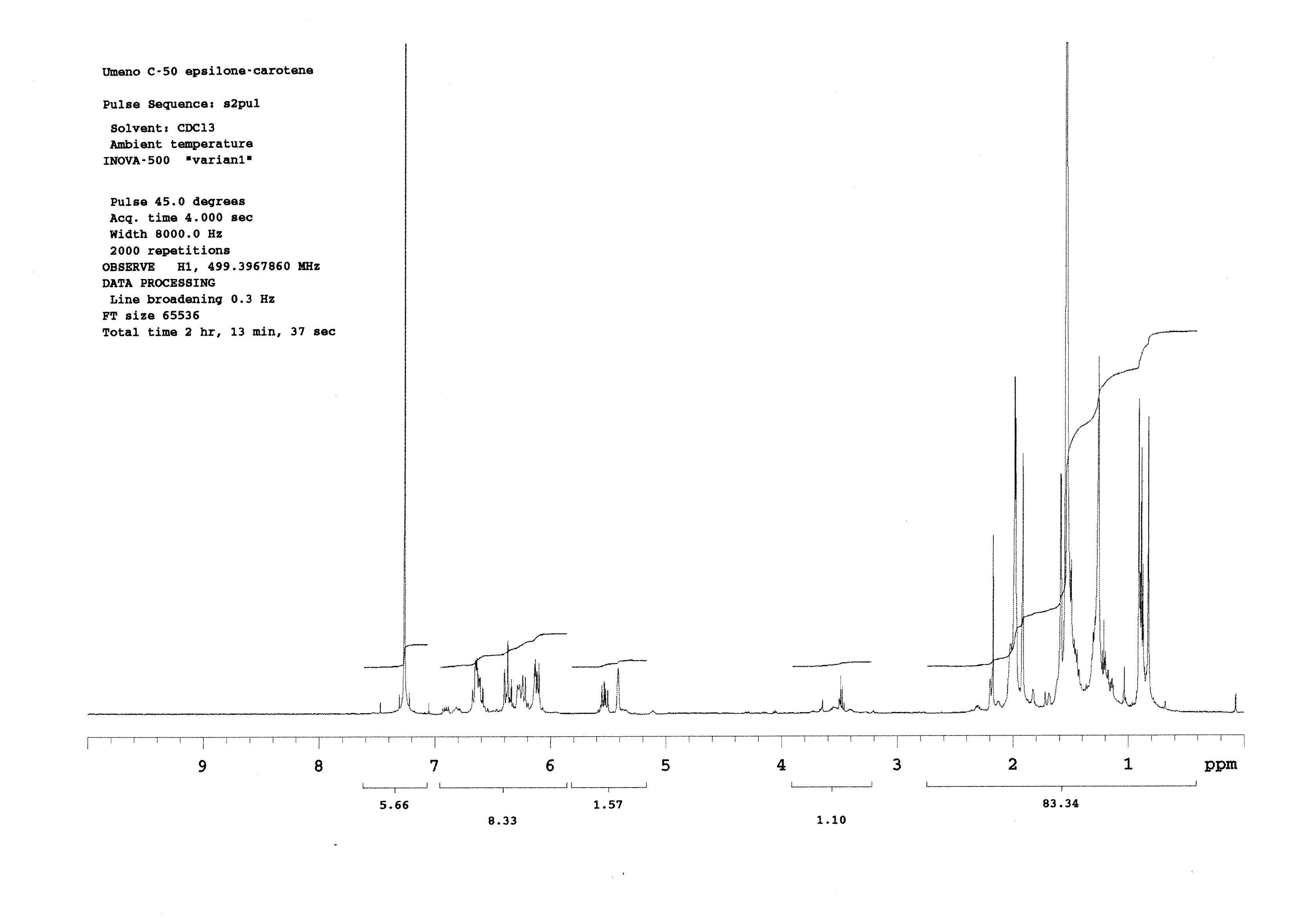

Supplement: S3 Fig — (JPG) [file pone.0216729.s005.jpg]

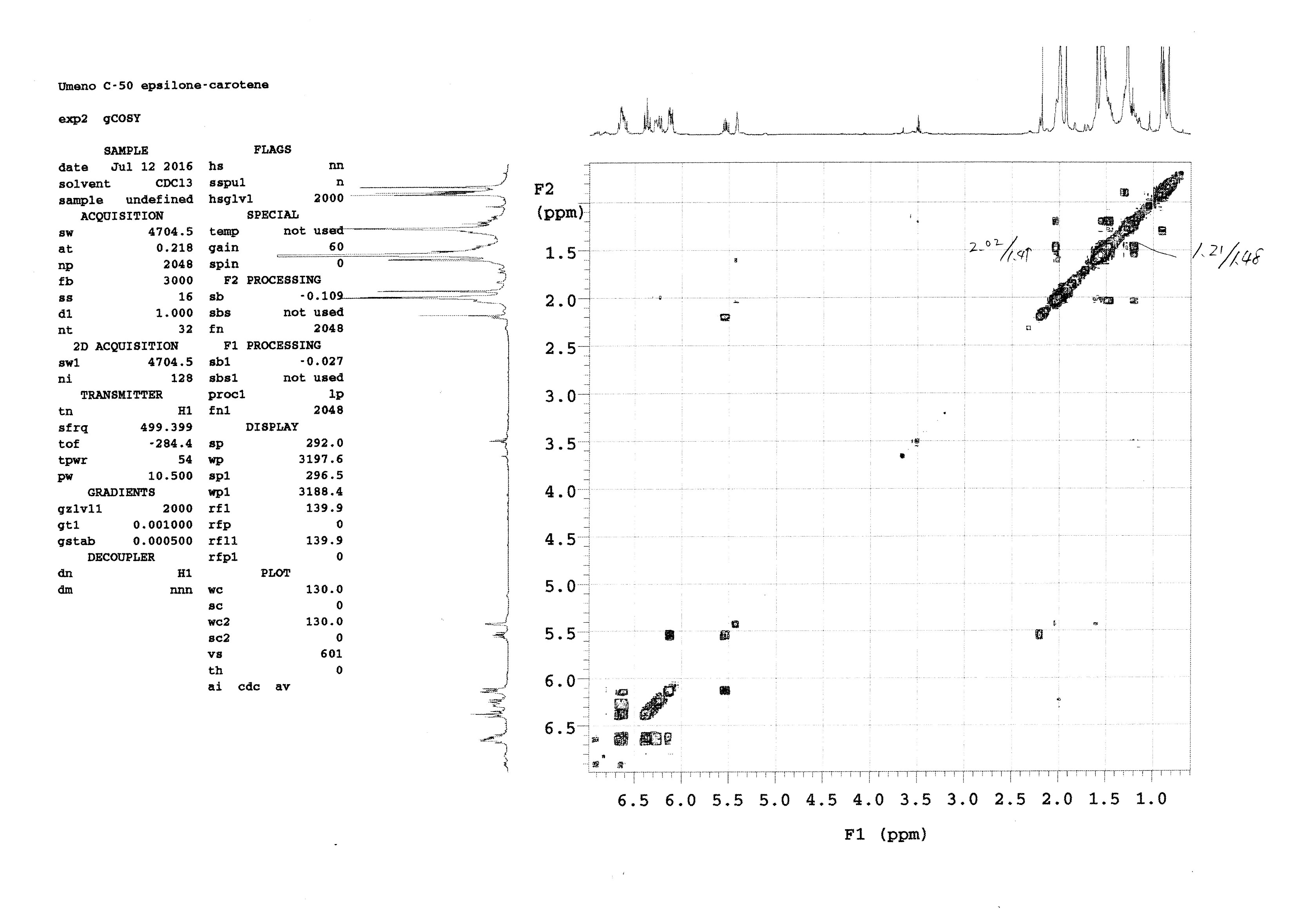

Supplement: S4 Fig — (JPG) [file pone.0216729.s006.jpg]

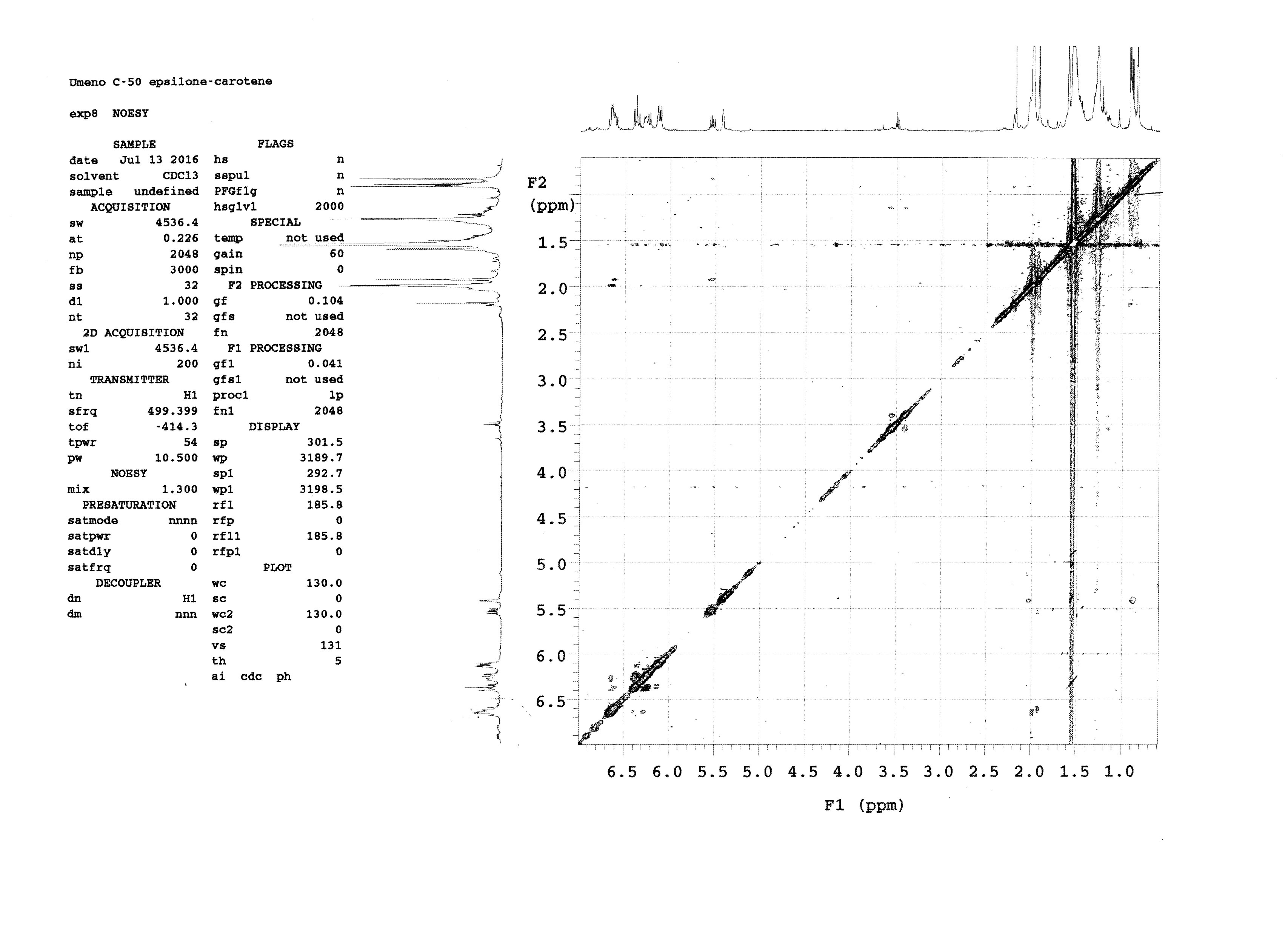

Supplement: S5 Fig — (JPG) [file pone.0216729.s007.jpg]

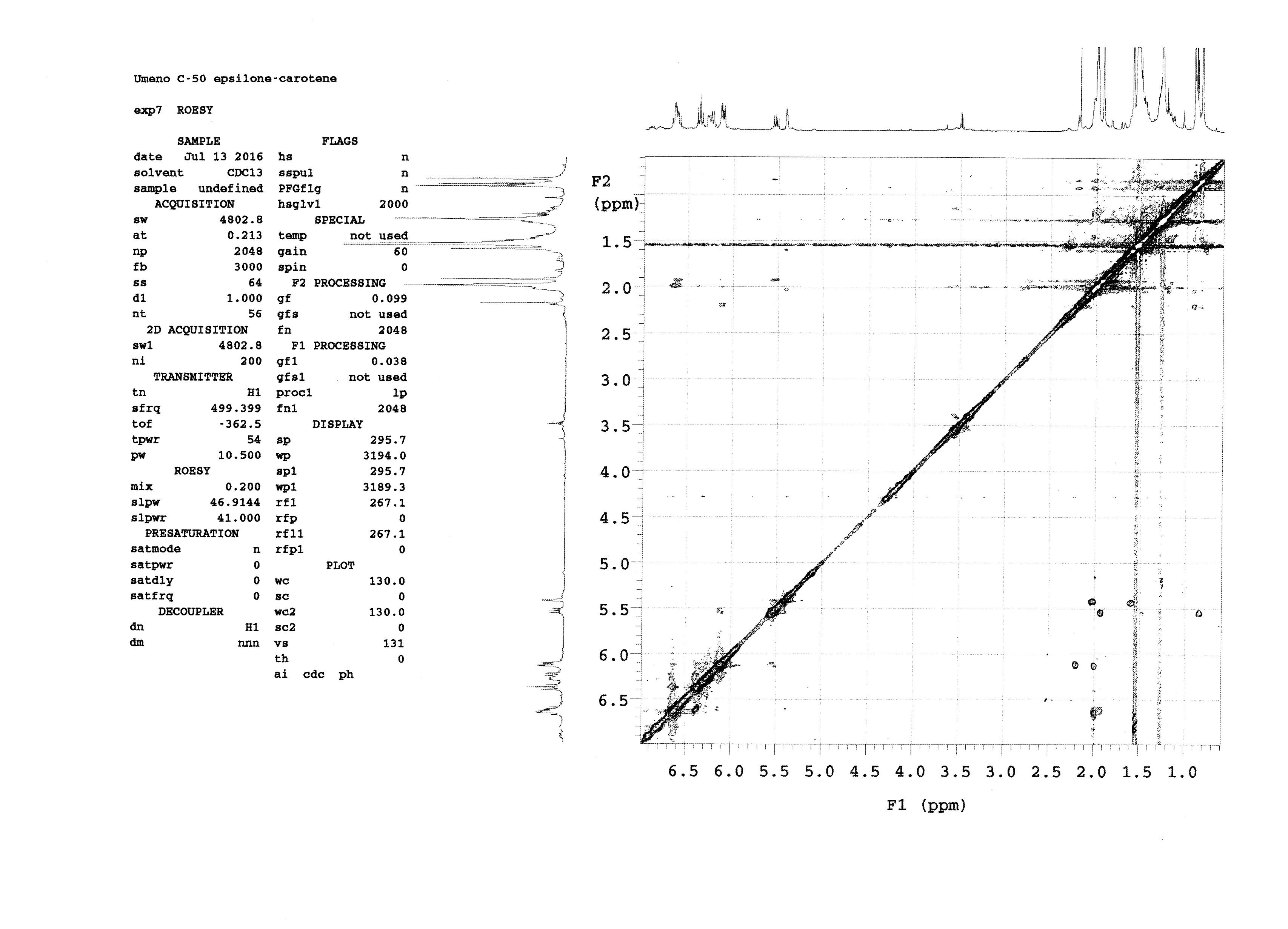

Supplement: S6 Fig — (JPG) [file pone.0216729.s008.jpg]

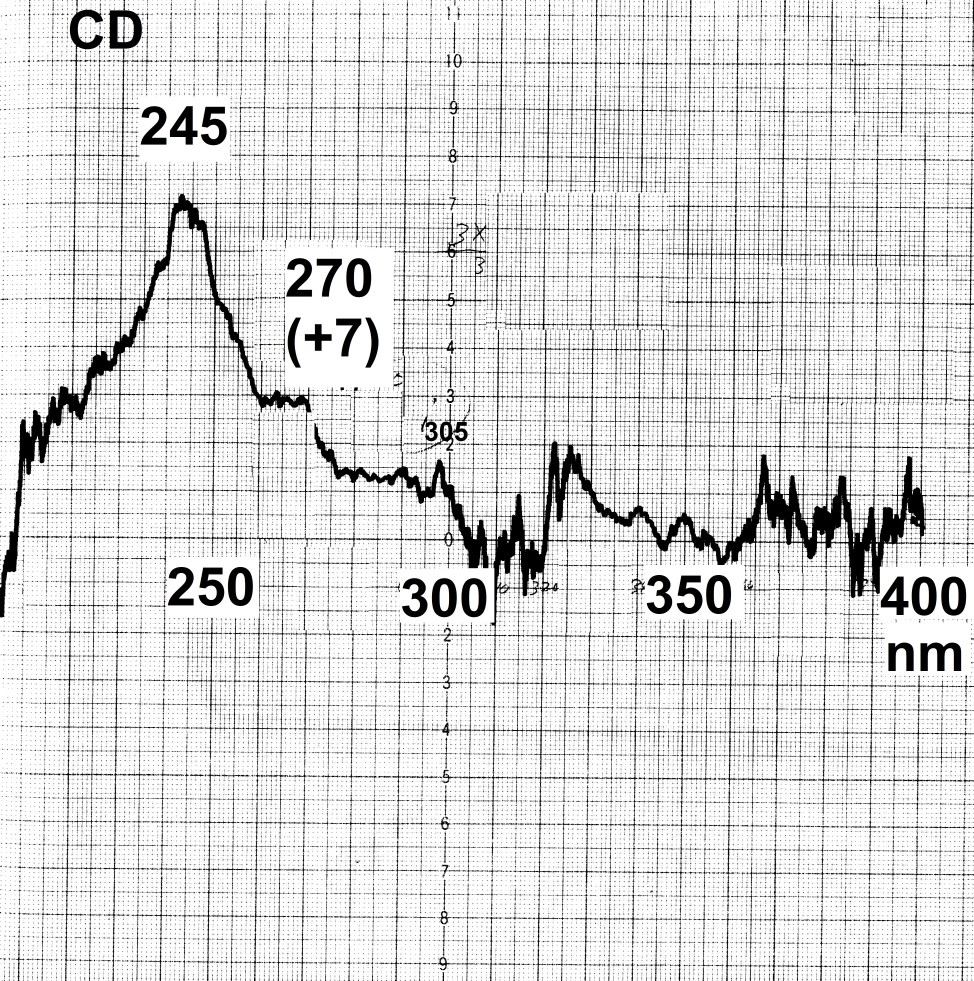

Supplement: S7 Fig — (JPG) [file pone.0216729.s009.jpg]
